# Biometric variability of inflorescence and flower traits among ex situ accessions of the neotropical oilseed palm *Acrocomia* Mart.

**DOI:** 10.1002/ece3.70053

**Published:** 2024-07-30

**Authors:** Catherine Meyer, Thomas Hilger, Kalcida Naomi Kuki, Sérgio Yoshimitsu Motoike, Georg Cadisch

**Affiliations:** ^1^ Hans‐Ruthenberg‐Institute for Tropical Agricultural Sciences University of Hohenheim Stuttgart Germany; ^2^ Department of Agronomy Federal University of Viçosa Viçosa Brazil

**Keywords:** *Acrocomia*, flower biometry, inflorescence architecture, oilseed palm, phenotypic diversity, taxonomy

## Abstract

The oilseed palm genus *Acrocomia* is suitable for sustainable oil production in South America. The high phenotypic diversity of wild populations poses a challenge for the delimitation of the genus. Comparing the inflorescence architecture, a first‐order panicle, and staminate and pistillate flower traits could be a valuable tool in resolving the taxonomic disarray. Thus, this study aims to characterize the differences in the inflorescence architecture and floral structures of three common and economically significant *Acrocomia* species: *A. aculeata*, *A. totai*, and *A. intumescens*. Biometric traits of the inflorescence architecture and floral structures of various *Acrocomia* accessions in an ex situ germplasm collection in Brazil were assessed. The unweighted pair group method with arithmetic mean (UPGMA) cluster analysis based on the Gower distance was used to measure dissimilarities between the individual plants of the accessions. To our best knowledge, this study provides the first evidence of the presence of second‐order rachillae in the genus *Acrocomia*. Evaluated traits showed a high level of variation within and between accessions, emphasizing the phenotypic diversity of the genus. The accessions of *A. totai* were distinguishable from those of the other two species by their inflorescence architecture and flower traits. The dissimilarities between *A. aculeata* and *A. intumescens* were not sufficient to differentiate both. In conclusion, the quantitative assessment of inflorescence and floral traits is a valuable tool for taxonomic resolution of the genus.

## Graphical abstract

Biometric evaluation of inflorescence and flower traits of the oilseed palm genus *Acrocomia* could contribute to the delimitation of the species and to the understanding of yield formation. This study identifies the presence of second‐order rachillae in *Acrocomia* and reveals a high variation in inflorescence architecture and floral structures among three species.

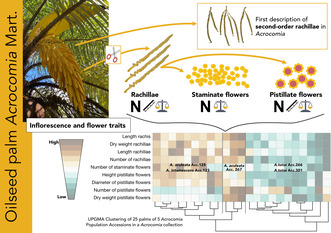

## 1 INTRODUCTION

The palm family (Arecaceae) includes 181 genera and more than 2600 species (Baker & Dransfield, 2016; Dransfield et al., 2008), which are easily recognized by their distinctive appearance in general, but also exhibit immense morphoanatomical diversity. The genus *Acrocomia* Mart. (subfamily Arecoideae, tribe Cocoseae) includes eight species (Flora e Funga Do Brasil, 2024; Lorenzi et al., 2010; POWO, 2024; Vianna, 2017; WFO, 2024), endemic to the semiarid and subhumid tropical regions of Central and South America (Dransfield et al., 2008; Lorenzi et al., 2010; Vianna, 2017). The *Acrocomia* palms are known under a range of common names depending on the region: e.g., macaúba, bocaíuva, macaíba, or grugru in Brazil; mbocayá in Argentina and Paraguay; coyol in Mexico to Costa Rica; and corozo or tamaco in Columbia. The three arborescent species *Acrocomia aculeata* (Jacq.) Lodd. ex Mart., *Acrocomia totai* Mart., and *Acrocomia intumescens* Drude are of great economic interest for vegetable oil production due to their wide geographical distribution (Colombo et al., 2018), and the quantity and quality of oil produced (Freitas de Lima et al., 2021; Montoya et al., 2016; Motoike et al., 2013; Pires et al., 2013).

While efforts have been made to domesticate *Acrocomia* (Cardoso et al., 2017; Montoya et al., 2016; Rosado et al., 2019), the lack of information on its phenotypic plasticity makes the delimitation of *Acrocomia* species (Díaz et al., 2021; Vianna et al., 2021) and consequently breeding and conservation programs more challenging (de Lima et al., 2018; Lanes et al., 2016; Mazzottini‐dos‐Santos et al., 2015). The taxonomic identification of the *Acrocomia* species relies heavily on mainly qualitatively assessed, morphological characteristics of various botanical components, including leaves (Vianna, Carmelo‐Guerreiro, et al., 2017), fruits (Madeira et al., 2024; Vianna et al., 2021; Vianna, Berton, et al., 2017), as well on stem morphology, general habit, and geographical distribution information (Díaz et al., 2021; Lorenzi et al., 2010). However, stem characteristics, such as diameter, shape, presence of spines and leaf scars, or persistence of leaf bases, can also be linked to changing growing conditions and plant age (Tomlinson, 1990; Tomlinson et al., 2011). In addition, the overlap in morphological traits is exacerbated by the high phenotypic diversity of wild populations (Abreu et al., 2012; Díaz et al., 2021; Lanes et al., 2016; Madeira et al., 2024; Oliveira et al., 2012). Consequently, species misidentifications of the wild populations are common as species‐related morphological differences are disregarded and viewed as phenotypic variations based on the environmental conditions, or vice versa (de Lima et al., 2018; Vianna et al., 2021).

There has been much disagreement among scientists on the taxonomic status of the *Acrocomia* species and the existing literature has not distinguished between species in a systematic way. For instance, *A. totai* and *A. intumescens* are considered either synonyms (Henderson, 1995; Lanes et al., 2015; Menezes Oliveira et al., 2013; Montoya et al., 2016) or ecotypes (De Lima et al., 2020; Machado et al., 2015; Madeira et al., 2024) of *A. aculeata*, or as distinct species (Díaz et al., 2021; Silva et al., 2020; Vianna et al., 2021; Vianna, Berton, et al., 2017; Vianna, Carmelo‐Guerreiro, et al., 2017). This contributes to a limited understanding of the economic potential of the species (de Lima et al., 2018; Vianna et al., 2021) and constraints in domestication programs and diversity conservation strategies (Hey et al., 2003; Morrison et al., 2009). Regarding the species uncertainty and a genus in need of taxonomic revision, we suggest considering the current taxonomic *Acrocomia* species as hypothetical taxon entities, to be confirmed or rejected by morphoanatomical and genetic data available in the present and future.

The knowledge on the structural variation of inflorescences, flower clusters, and flowers is scarce (Vargas‐Carpintero et al., 2021) and focuses mainly on *A. aculeata* (Mazzottini‐dos‐Santos et al., 2015), and remains limited for other *Acrocomia* species and intraspecific populations. *Acrocomia* palms are synchronously monoecious and protogynous, that is, the pistillate flowers are receptive before the anthesis of the staminate flowers (Mazzottini‐dos‐Santos et al., 2015; Scariot et al., 1991). The inflorescence, an interfoliar panicle with branching of the first order, consists of a rachis carrying several hundred rachillae. The rachillae are extended and bear each distally a large number of staminate flowers present in pairs, flower clusters referred to as dyads, or solitary (Henderson, 1995; Mazzottini‐dos‐Santos et al., 2015; Scariot et al., 1991). At the base of the rachillae, the few pistillate flowers are represented in triads, a floral cluster of a central pistillate flower flanked by two staminate flowers (Henderson, 1995; Mazzottini‐dos‐Santos et al., 2015; Scariot et al., 1991). The floral morphology of the pistillate and staminate flowers shows a pronounced dimorphism. The globose pistillate flowers, with connate and overlapping petals, are larger than the elongated trimerous staminate flowers (Mazzottini‐dos‐Santos et al., 2015).

A thorough morphological characterization of the inflorescences and floral structures on the species and population level would facilitate an understanding of yield formation, serve as a basis for domestication initiatives, and provide important information for taxonomic, molecular, genomic, ecological, and bioeconomic studies. Therefore, we aimed to characterize interspecies and intraspecies phenotypical differences in the inflorescence architecture and floral structures of *A. aculeata*, *A. totai*, and *A. intumescens*. The study sought to answer the following questions: (1) How do the species differ in their quantitatively measurable inflorescence and flower traits? (2) Are vestigial floral structures present, and in which species are they occurring? (3) Can the characterization of floral and inflorescence traits contribute to the taxonomic resolution between these three *Acrocomia* species? We assessed six accessions of wild populations located in an *Acrocomia* ex situ conservation collection for their inflorescence, rachillae, and flower biometry. To our best knowledge, this study is the first to report the presence of second‐order rachillae in the genus *Acrocomia* and to give a detailed insight into the variation of inflorescence architecture present between populations of *Acrocomia*.

## 2 MATERIALS AND METHODS

### 2.1 Study site and accessions of botanical material

This study was conducted at the living *Acrocomia* spp. Germplasm Bank, named BAG‐Macaúba (register number 084/2013 SECEX/CGEN), of the University of Viçosa, Brazil. This ex situ collection of around 300 *Acrocomia* populations originating from various regions of South America is located in Araponga (latitude 20°40′1′′ S; longitude 42°31′15′′ W) in the South‐east of the federal state Minas Gerais in Brazil. The climate in Araponga, located at 1200 m above mean sea level, is subtropical with a dry winter and a warm summer (Cwb) according to the Köppen Climate Classification (Alvares et al., 2013). The natural vegetation is Atlantic rainforest (in Portuguese, Mata Atlântica).

Within the *Acrocomia* spp. Germplasm Bank BAG‐Macaúba, we selected accessions of wild populations (hereafter only referred to as accessions) based on the following criteria: (A) their region of origin using the Global Positioning System (GPS) data provided by the BAG‐Macaúba database; (B) the presence of inflorescence or fruit bunches in November 2018 to ensure producing palms; (C) the number of plants per accession in the collection (at least 3); and (D) a palm height below 7 m for labor safety and time. Finally, we retained six accessions with a total of 31 palms (Table 1).

**TABLE 1:** List of the six accessions of *Acrocomia* assessed in the Macaúba Active Germplasm Bank (BAG‐Macaúba).

Accession designation	BAG‐Macaúba Accession BAG‐ID	Year of planting	Species	Number of palms assessed	Region of origin	Köppen climate in region of origin (Alvares et al., 2013)
INT123	BGP 123	2011	*Acrocomia intumescens*	3	Western Paraíba	As
ACL267	BGP 267	2013	*A. aculeata*	5	Northern Minas Gerais	As
ACL125	BGP 125	2011	*A. aculeata*	6	Midsouthern Minas Gerais	Cwb
ACL270	BGP 270	2013	*A. aculeata*	6	Midwestern Minas Gerais	Cwa
TOT266	BGP 266	2013	*A. totai*	6	Central Mato Grosso do Sul	Am
TOT301	BGP 301	2013	*A. totai*	5	Paraguay	Cfa

*Note*: The designation consists of the species epithet and accession number in the BAG‐Macaúba. The regions of origin are deposited in the BAG‐Macaúba database and the Köppen climate was excerpted from the supplemental material of Alvares et al. (2013).

**FIGURE 1:**
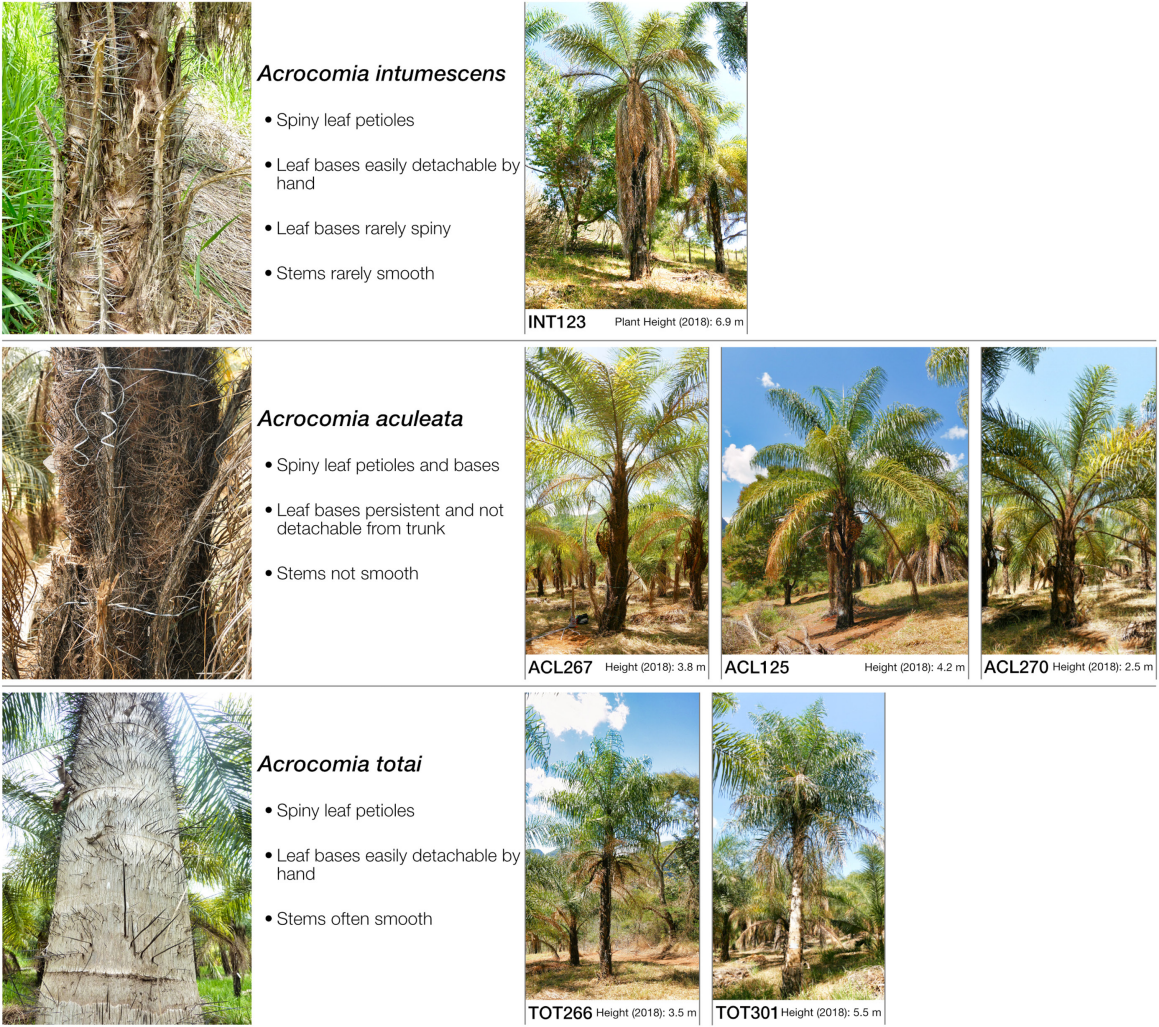
Left side: Stem morphology of *Acrocomia intumescens*, *A. aculeata*, and *A. totai*. The morphology of the stem is currently the most important trait for species distinction. Right side: One plant of each accession (including the height (soil to crown base) of that specific plant in December 2018).

We assessed visually the vegetative traits of the stem (stem swelling, leaf base persistence, and spine coverage; Figure 1), leaves, and fruit size to assign the species to the accessions using the taxonomic key of Lorenzi et al. (2010) in combination with the biogeographic origin of the accessions. Hereafter, we refer to each accession with their species’ epithet, abbreviated as ACL, TOT, and INT, for *A. aculeata*, *A. totai*, and *A. intumescens*, respectively, followed by their accession number (BAG‐ID) in the BAG‐Macaúba (see Table 1).

### 2.2 Evaluated traits and the study time frame

We evaluated a total of nine morphological characteristics descriptive for inflorescence architecture (length of rachis; number of rachillae; and length of rachillae), rachillae (dry weight of rachillae), and flower traits (number of pistillate flowers; number of staminate flowers; height of pistillate flowers; diameter of pistillate flowers; and dry weight of pistillate flowers).

The length of rachillae, and rachillae and flower traits were measured during the flowering periods of 2019 and 2021. In 2020, a detailed sampling for the evaluation of rachillae and flower traits was not possible due to travel restrictions during the COVID‐19 pandemic.

Additionally, the number of inflorescences per palm tree was counted and labeled during the flowering periods of 2019, 2020, and 2021. As the assessment of rachis length and number of rachillae was unfeasible during the flowering periods, these measurements were taken after harvesting the fruit bunches in 2021 and 2022. The fruit bunches were detached from the palms by severing the peduncle with a cutting device.

### 2.3 Inflorescence and flower traits

Every other day during the flowering periods of 2019 and 2021, we surveyed the 31 individuals for any open inflorescences and sampled a total of nine rachillae from each newly opened inflorescence. Three rachillae were collected, respectively, from the basal, middle, and apical third of the inflorescences. To assess the variation in floral structures, we recorded for each rachilla the number of the following structures: triads and number of flanking staminate flowers present; staminate and pistillate dyads; staminate flowers with a well‐developed, infertile carpel; rachilla ramifications; and triple and single staminate flowers only when present between the pistillate flowers. In addition, the order of occurrence of these floral structures was determined from the most basal end of the rachilla to the apex. The most basal floral structure was assigned “Position 1” and the character of the floral structure was recorded. The subsequent floral structure was designated “Position 2” and its character was also recorded. This was repeated for each subsequent floral structure until the singular staminate flowers of the apex were reached.

Afterward, all floral structures were separated from the rachillae. The height and diameter of the pistillate flowers were determined with a digital sliding scale. The diameter of the pistillate flowers was measured along the axis parallel to the stalk (Diameter 1) and the axis perpendicular to the stalk (Diameter 2). The total length of the rachillae was determined with a tape measure. Furthermore, it was noted if the staminate flowers already started anthesis at the time of sampling. In addition, the lengths of the apical region, containing only the staminate flowers, and the basal region, containing the pistillate and staminate dyads, were measured. Fresh and dry weights of each rachilla, pistillate flower, and the total bulk of staminate flowers per rachilla were determined. All botanical materials were dried at 65°C in a ventilated oven to constant weight. Lastly, the dried staminate flowers were counted.

After the harvest of the fruit bunches, the length of the rachis was measured with a measuring tape. The rachillae were separated from the rachis with pruning shears. The rachillae of the individual fruit bunches were grouped according to the number of ramifications and then counted.

### 2.4 Statistics and picture editing

The inflorescence architecture and flower traits were statistically described with the following measures of position and dispersion: Median, interquartile ranges (IQRs), probability density, and median absolute deviation (MAD).

Spearman's rank correlation coefficient was used to assess the association between the following traits: rachis length, number of rachillae, rachillae dry weight, rachillae length, length of proportions of rachillae with pistillate and staminate flowers, number of pistillate and staminate flowers per rachillae, and dry weight, height and diameter of pistillate flowers.

**FIGURE 2:**
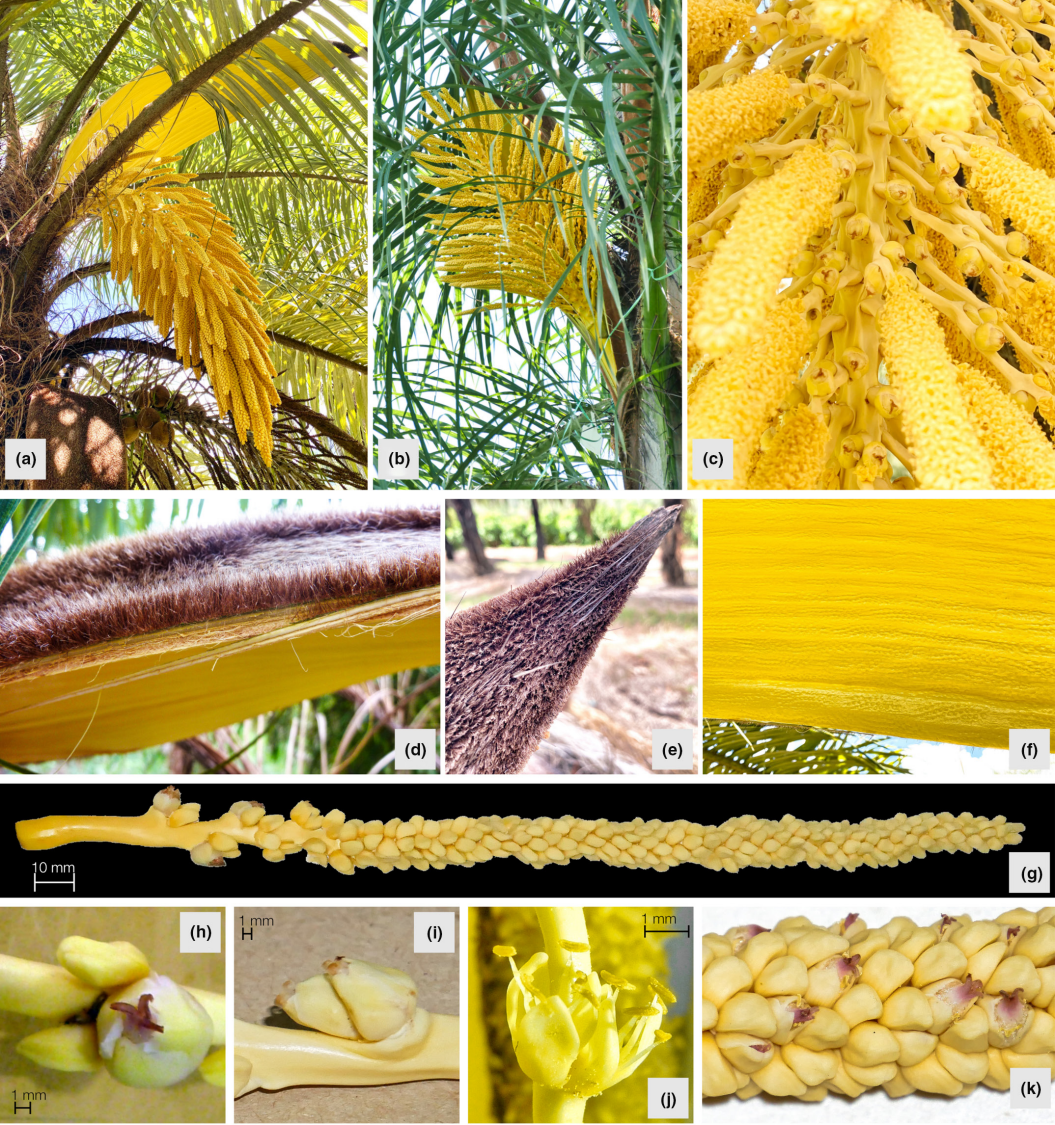
An overview of the inflorescence and floral structure of *Acrocomia* species. (a) Inflorescence of *A. aculeata*. (b) Inflorescence of *A. totai*. (c) Detailed view of the rachis and attached rachillae (*A. aculeata*). (d–f) Details of the bract showing the abaxial dense layer of short brown trichomes with the occasional aculeus, and bright yellow and glabrous adaxial surface. (g) Rachillae. (h) Triad. (i) Pistillate dyad. (j) Staminate dyad. (k) Staminate flowers with a well‐developed infertile carpel in the apical end of rachillae.

**FIGURE 3:**
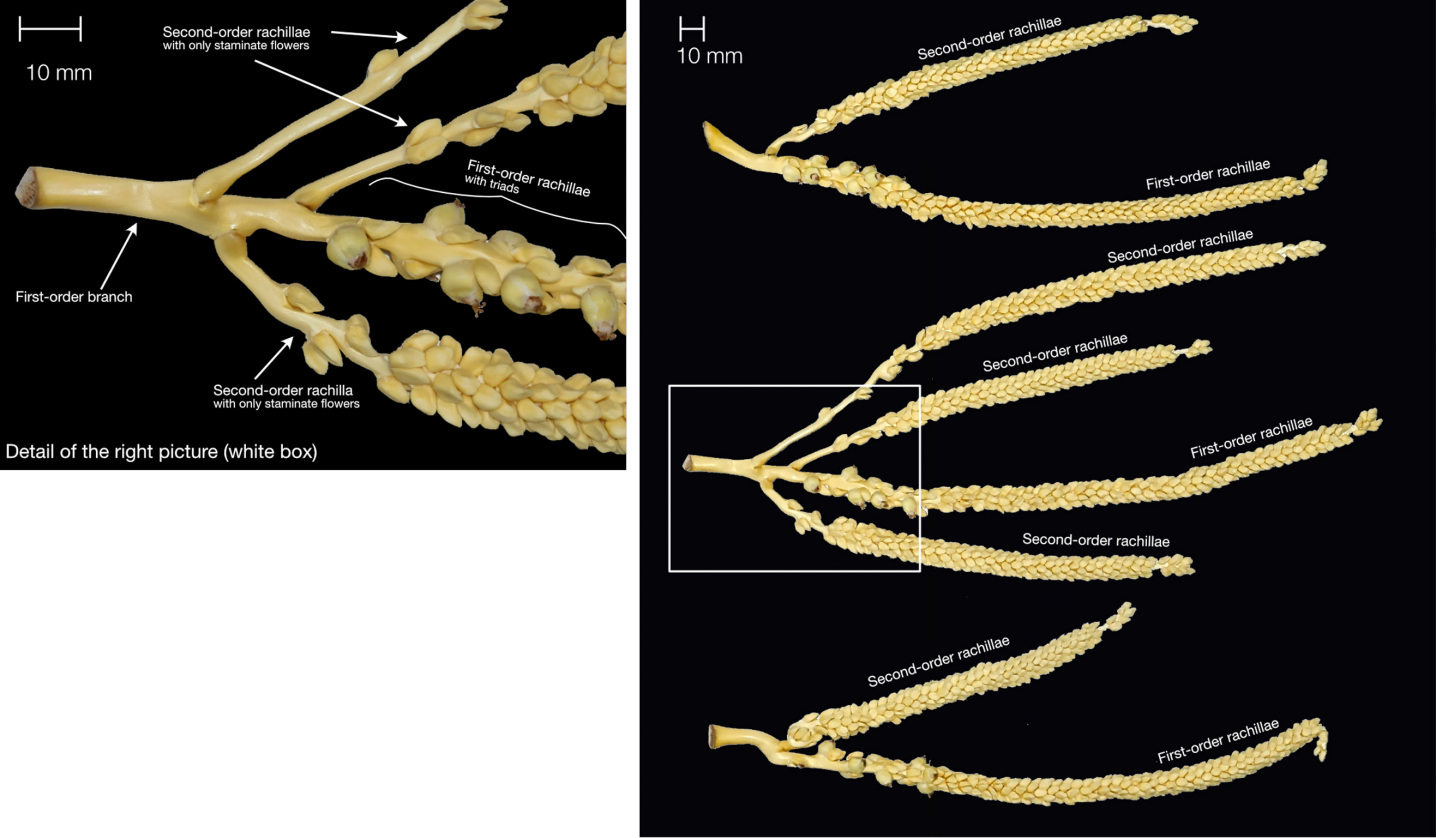
Second‐order rachillae in *Acrocomia totai*. Right: Branches with 1 or 3 second‐order rachillae. Left: Detail showing the branching of the second‐order rachillae off the first‐order branch before the start of the flower‐bearing first‐order rachillae.

All descriptive measures and Spearman's rank correlation coefficients were calculated using JMP 17 software, Version 17.1.0 (JMP Statistical Discovery LLC, USA). To evaluate the shared inflorescence architecture and flower traits between the individual palm trees, we subjected the traits to cluster analysis using the Gower distance and the UPGMA algorithm. The analyses were performed using the distance and cluster procedure in SAS software, Version 9.4 (SAS Institute Inc., USA). All pictures were edited using Luminar 4 software, Version 4.3.5 (Skylum Software, USA) to correct exposure and contrast across the entire image. In the picture g in Figure 2 and the pictures in Figure 3, the background (a white tile floor) was edited out using Affinity Photo, Version 2.3.1 (Serif (Europe) Ltd., UK). No changes in tint, tone, shade, and color hue were made in all pictures.

## 3 RESULTS

### 3.1 Inflorescence architecture and rachillae biometry

While emerging interfoliar, the young inflorescences were enclosed by a large woody peduncular bract. The bracts split abaxially along longitudinal slits, releasing inflorescences with an intense sweet odor. The abaxial face of the persistent bracts was covered in a dense layer of short brown trichomes with the occasional aculeus (Figure 2d–e). Their adaxial surface was bright yellow and glabrous (Figure 2f).

**FIGURE 4:**
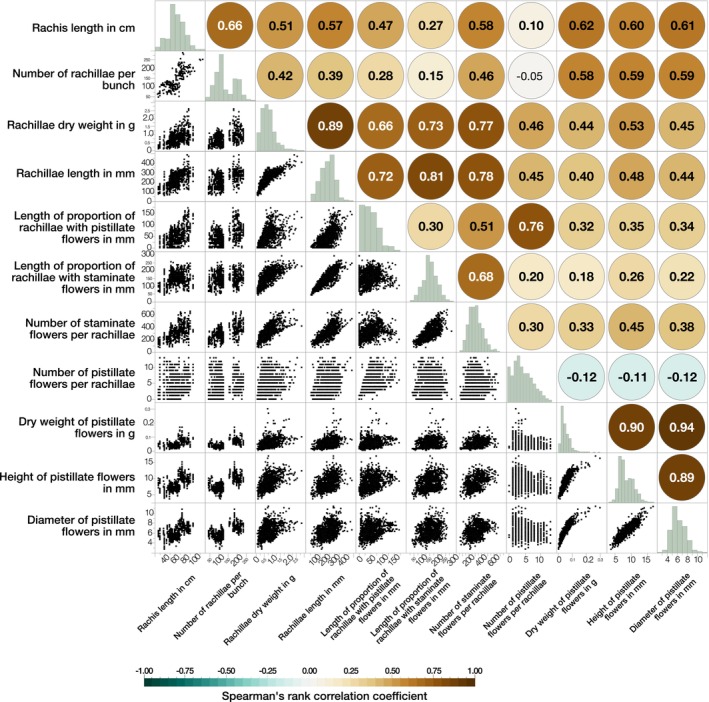
Scatterplot matrix illustrating the pairwise relationship between the inflorescence architecture and flower traits of *Acrocomia*, along with the corresponding Spearman's rank correlation coefficient (rho). Statistically significant correlations (*p* < .05) are highlighted in bold. The histograms display the distributions.

**FIGURE 5:**
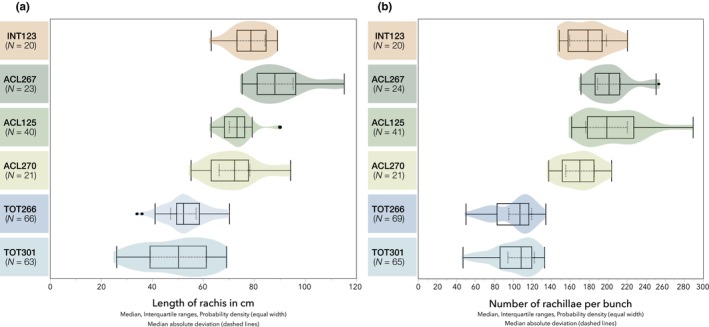
Biometric characteristics of the *Acrocomia* inflorescence architecture. Length of rachis in cm (a). Number of rachillae (b). Median, quartiles, probability density (equal width), and median absolute deviation (MAD) of a total *N* of inflorescences of both flowering seasons of 2019/2020 and 2020/2021 at the BAG‐Macaúba, Araponga, MG, Brazil.

The inflorescences of all palms were consistent in their structure, being composed of a main rachis and shorter first‐order rachillae, bearing the flowers. The rachis length was positively correlated with the number of rachillae per inflorescence (*r*_s_ = 0.66, *p* < .0001; Figure 4). The inflorescences of TOT266 and TOT301 were visually distinguishable from the inflorescences of the ACL and INT accessions (Figure 2a–b). The shorter rachis (Figure 5a) with the lower number of rachillae (Figure 5b) were suberect between the leaves. The short rachillae (Table 2) gave the panicle a more rigid appearance. The inflorescences of INT123 were visually not discriminable from ACL125 and ACL267. They had a comparable length of the rachis and number of rachillae. The panicle had a pendulous appearance with the long rachillae dangling from the rachis.

**TABLE 2:** Biometric characteristics of the rachillae.

Species	*A. intumescens*	*A. aculeata*		*A. totai*	
Accession	INT123	ACL267	ACL125	TOT266	TOT301
*N* of assessed rachillae	81	132	308	493	399
Dry weight in g					
Median	1.11	0.99	0.87	0.59	0.42
Minimum	0.63	0.28	0.35	0.16	0.08
Maximum	2.06	2.87	2.35	1.46	1.32
IQ range	0.68	0.81	0.43	0.43	0.41
MAD	0.33	0.36	0.21	0.22	0.19
Total length in mm					
Median	265	295.5	276	227	165
Minimum	172	94	171	86	60
Maximum	357	478	376	355	344
IQ range	66.5	82	58.75	81.5	86
MAD	33	39.5	28	43	40
Proportion of length bearing pistillate flowers in %					
Median	19.3	35.6	20.5	18.0	19.0
Minimum	7.4	5.7	0.0	0.0	0.0
Maximum	33.3	66.3	42.4	38.8	39.5
IQ range	8.7	18.0	14.4	16.1	16.9
MAD	4.4	9.0	6.9	7.4	7.7
Proportion of length bearing staminate dyads in %					
Median	4.6	4.5	7.2	10.1	11.4
Minimum	0.9	0.0	1.7	0.0	0.0
Maximum	18.3	18.6	35.5	30.7	43.9
IQ range	6.5	3.0	5.4	7.3	10.8
MAD	2.3	1.4	2.4	3.6	4.8
Proportion of length bearing single staminate flowers in %					
Median	56.7	42.9	54.3	60.8	59.4
Minimum	42.6	30.7	37.1	43.1	41.1
Maximum	73.2	68.1	86.6	96.5	95.9
IQ range	8.6	12.5	11.1	10.5	12.8
MAD	4.2	5.7	5.6	4.9	6.6
Dry matter in %					
Median	30.0	30.7	28.5	29.7	32.1
Minimum	26.3	26.1	22.0	24.2	21.1
Maximum	43.2	40.3	43.1	54.9	79.7
IQ range	4.7	3.4	4.1	5.7	6.6
MAD	1.8	1.5	1.9	2.8	3.3

*Note*: Median, upper and lower limit, interquartile range (IQ range), and median absolute deviation (MAD) of a total *N* of rachillae assessed in both flowering seasons of 2019/2020 and 2021/2022 at the BAG‐Macaúba, Araponga, MG, Brazil.

### 3.2 Floral biometry

The rachillae displayed an androgynous nature with an overabundance of staminate flowers in comparison to pistillate flowers with a ratio of staminate:pistillate flowers from 51:1 to 133:1 (Table 3). The number of staminate flowers varied between 68 and 666, with some differences observed between the accessions. ACL267, INT123, and ACL125 possessed a higher number of staminate flowers compared to TOT266 and TOT301, which was attributed to the elongated apical region of the rachillae documented in the former three accessions. The Spearman correlation analysis also revealed a significant positive correlation between the number of staminate flowers and the length of the rachillae (*r*_s_ = 0.78, *p* < .0001) and the proportion of length bearing the staminate flowers (*r*_s_ = 0.68, *p* < .0001).

**TABLE 3:** Number of rachillae without pistillate flowers, and number of staminate and pistillate flowers per rachillae.

Species	*A. intumescens*	*A. aculeata*		*A. totai*	
Accession	INT123	ACL267	ACL125	TOT266	TOT301
*N* of rachillae assessed	81	131	307	462	394
*N* of rachillae with pistillate flowers	81	130	278	399	341
*N* of rachillae without pistillate flowers	0	1	29	63	53
Number of pistillate flowers per rachilla					
Median	3	6	3	5	4
Minimum	2	0	0	0	0
Maximum	6	13	8	13	13
IQ range	1	4	2	5	5
MAD	1	2	1	3	3
Number of staminate flowers per rachilla					
Median	433	315	403	296	210
Minimum	290	108	198	122	68
Maximum	624	529	666	454	341
IQ range	99	147	141	87	75
MAD	50	73	72	43	39
Number of staminate flowers per pistillate flowers					
Median	133	51	123	58	51
Minimum	60	27	65	23	12
Maximum	263	143	589	338	331
IQ range	61	27	70	55	61
MAD	29	12	32	20	26

*Note*: Median, upper and lower limit, interquartile range (IQ range) and median absolute deviation (MAD) of a total *N* of rachillae assessed in both flowering seasons of 2019/2020 and 2021/2022 at the BAG‐Macaúba, Araponga, MG, Brazil.

**FIGURE A1:**
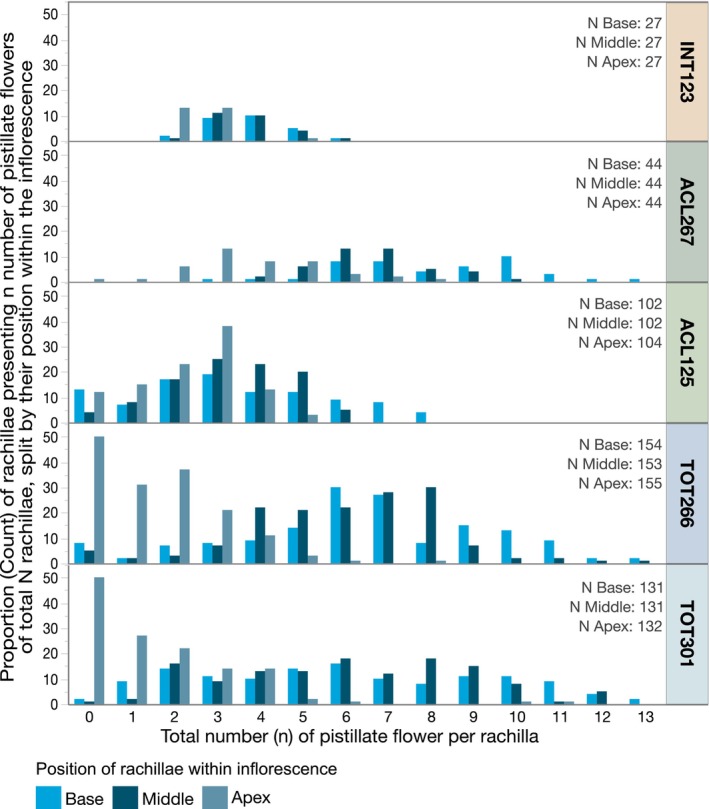
Absolute frequency of the number of pistillate flowers per rachilla of a total *N* of rachillae split by the position of the rachillae within the inflorescence (Base, Middle, Apex), assessed in both flowering seasons of 2019/2020 and 2021/2022 at the BAG‐Macaúba, Araponga, MG, Brazil.

ACL267, TOT266, and TOT301 exhibited the greatest range in the number of pistillate flowers per rachilla. Rachillae with only staminate flowers and no pistillate flowers were prevalent in TOT266 and TOT301, and somewhat frequent in ACL125, mainly in the apical third of the inflorescences (Figure A1). A common trait shared by all palms was the decrease in the number of staminate and pistillate flowers, as well as the length of the rachillae, from the base to the apex of the inflorescence (Figure A1, Tables A1 and A2, respectively, for a number of pistillate flowers, number of staminate flowers, and length of rachillae).

Visually, the pistillate flowers of TOT266 and TOT301 were distinguishable from those of INT123, ACL267, and ACL125 due to their size (Table 4), shape, and color. The pistillate flowers of TOT266 and TOT301 were small, globose, and yellow‐greenish in color. In comparison, the pistillate flowers of *A. intumescens* and *A. aculeata* were egg‐shaped, larger, and heavier with a light‐yellow hue. The number of pistillate flowers showed a weak negative correlation with the pistillate flower dry weight (*r*_s_ = −0.12, *p* < .0001), the pistillate flower height (*r*_s_ = −0.11, *p* < .0001), and the diameter (*r*_s_ = −0.12, *p* < .0001). INT123 and ACL125 had a wider spacing of pistillate flowers within the rachillae, which was related to longer rachillae and fewer pistillate flowers. While ACL267 had similar numbers of pistillate flowers to TOT266 and TOT301, it also exhibited wider spacing along with a higher proportion of rachillae length‐bearing pistillate flowers.

**TABLE 4:** Biometry of pistillate flowers.

Species	*A. intumescens*	*A. aculeata*		*A. totai*	
Accession	INT123	ACL267	ACL125	TOT266	TOT301
*N* of pistillate flowers assessed	273	822	880	2157	1788
Dry weight of pistillate flowers in mg					
Median	75	75	70	35	31
Minimum	31	31	23	13	4
Maximum	179	133	203	79	101
IQ range	39	35	26	13	25
MAD	19	16	13	6	12
Height of pistillate flowers in mm					
Median	10.3	9.7	10.5	7.1	6.9
Minimum	6.8	4.7	6.4	3.1	2.5
Maximum	14.7	14.7	16.7	10.5	11.7
IQ range	2.1	2.0	1.8	1.3	2.0
MAD	1.0	1.0	0.9	0.6	1.0
Diameter of pistillate flowers in mm					
Median	7.1	7.1	7.6	5.6	5.3
Minimum	4.8	4.6	5.2	3.3	2.2
Maximum	9.1	9.3	10.7	8.2	7.9
IQ range	1.4	1.3	1.1	0.9	1.4
MAD	0.7	0.6	0.6	0.4	0.7
Ratio Diameter 1 to Diameter 2					
Median	1.1	1.2	1.1	1.2	1.1
Minimum	0.7	0.4	0.7	0.5	0.5
Maximum	2.2	2.1	2.4	2.2	2.5
IQ range	0.2	0.3	0.2	0.3	0.2
MAD	0.1	0.1	0.1	0.1	0.1
Dry matter of pistillate flowers in %					
Median	25.6	23.8	20.7	24.3	23.7
Minimum	20.0	17.3	12.2	13.8	11.9
Maximum	31.6	33.2	31.7	45.2	35.5
IQ range	4.3	2.6	3.8	3.8	3.7
MAD	2.2	1.3	1.8	1.9	1.8

*Note*: Median, upper and lower limit, interquartile range (IQ range), and median absolute deviation (MAD) of a total *N of* rachillae assessed in both flowering seasons of 2019/2020 and 2021/2022 at the BAG‐Macaúba, Araponga, MG, Brazil.

### 3.3 Sequence and frequency of floral structures

**FIGURE 6:**
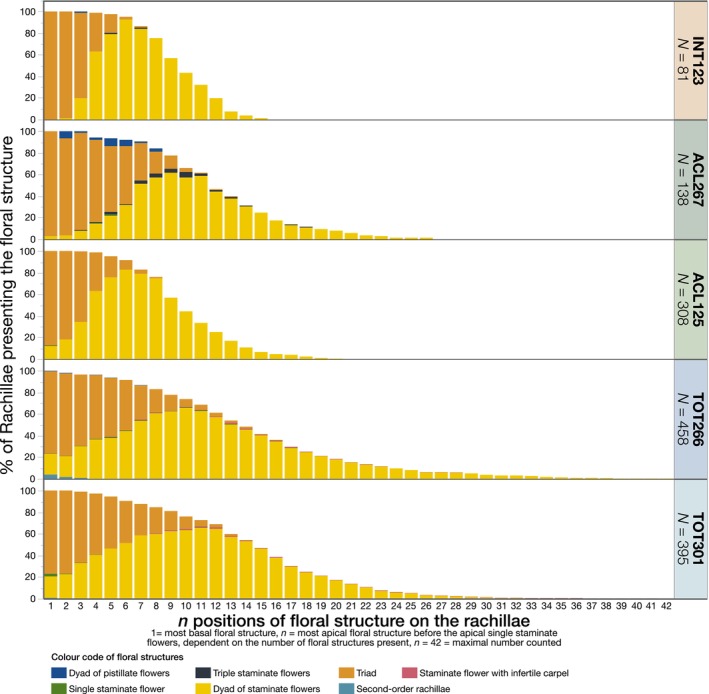
Sequence of floral structures along the rachillae from the basal end to the apical single staminate flower end of *N* rachillae assessed in both flowering seasons of 2019/2020 and 2021/2022 at the BAG‐Macaúba, Araponga, MG, Brazil.

The floral structures along the rachillae are displayed in Figure 6, showcasing the sequence from the basal end to the first single staminate flower at the apical end. The studied rachillae generally followed the sequence of floral structures as described for *Acrocomia*‐bearing triads at their basal end and staminate flowers toward the tip (Figure 2g‐j). However, some deviations from this pattern were observed, such as dyads between pistillate flowers and the absence of pistillate flowers altogether, not uncommon for Arecoideae.

Notably, several unusual variations were observed. For instance, dyads of pistillate flowers (Figure 2i) were found in 28 out of 132 rachillae assessed in ACL267 (Table 5a). In TOT266 and INT123, dyads of pistillate flowers were also present but a rare occurrence. Staminate flowers were present not only as dyads or flanking the pistillate flowers in triads, but also as singlets and in triplets (Figure 6). Only TOT266 and TOT301 displayed staminate flowers with a well‐developed infertile carpel (Table 5b and Figure 2k). This variation was observed in the triads and staminate dyads, and at the apical end of individual staminate flowers.

**TABLE 5:** (a) Frequency of occurrence of pistillate dyads and (b) staminate flowers with a well‐developed infertile carpel of a total *N* of rachillae assessed in both flowering seasons of 2019/2020 and 2021/2022 at the BAG‐Macaúba, Araponga, MG, Brazil. (c) Relative frequency of second‐order rachillae of a total *N* of rachillae assessed in both harvest seasons of 2020/2021 and 2021/2022.

Species	Accession	*N* rachillae	Total number	Minimal number present in 1 rachilla	Maximal number present in 1 rachilla	Number of rachillae presenting
(a) Occurrence of dyads of pistillate flowers						
*A. intumescens*	INT123	81	1	1	1	1
*A. aculeata*	ACL267	132	39	1	3	28
	ACL125	308	0	0	0	0
*A. totai*	TOT266	462	7	1	1	7
	TOT301	394	0	0	0	0
(b) Occurrence of staminate flowers with a well‐developed infertile carpel						
*A. intumescens*	INT123	81	0	0	0	0
*A. aculeata*	ACL267	132	0	0	0	0
	ACL125	308	0	0	0	0
*A. totai*	TOT266	462	86	1	9	33
	TOT301	394	41	1	19	9

(c) Relative frequency (%) of ramified rachillae grouped by *N* of second‐order rachillae									
Number of second‐order rachillae			0	1	2	3	4	5	6
Species	Accession	*N* Rachillae							
*A. intumescens*	INT123	3552	100	0	0	0	0	0	0
*A. aculeata*	ACL267	4868	100	0	0	0	0	0	0
	ACL125	8385	100	0	0	0	0	0	0
	ACL270	3543	100	0	0	0	0	0	0
*A. totai*	TOT266	7027	95.26	2.73	1.32	0.51	0.10	0.04	0.03
	TOT301	6754	99.90	0.10	0	0	0	0	0

The most noteworthy deviation was the occurrence of second‐order rachillae in *A. totai* (Table 5c and Figure 3). These rachillae were observed in the most basal positions of the first‐order branches, preceding the pistillate flowers of the rachilla. While a single second‐order rachilla was the most common, up to 6 were counted. The second‐order rachillae were sunken into depressions in the first‐order branch in a similar manner to the flowers. The second‐order rachillae displayed the usual pattern observed in *Acrocomia* rachillae, with single staminate flowers at the tip and pistillate flowers at the base, although 77.4% of the second‐order rachillae (data not shown) were not bearing any pistillate flowers.

### 3.4 Cluster analysis

**FIGURE 7:**
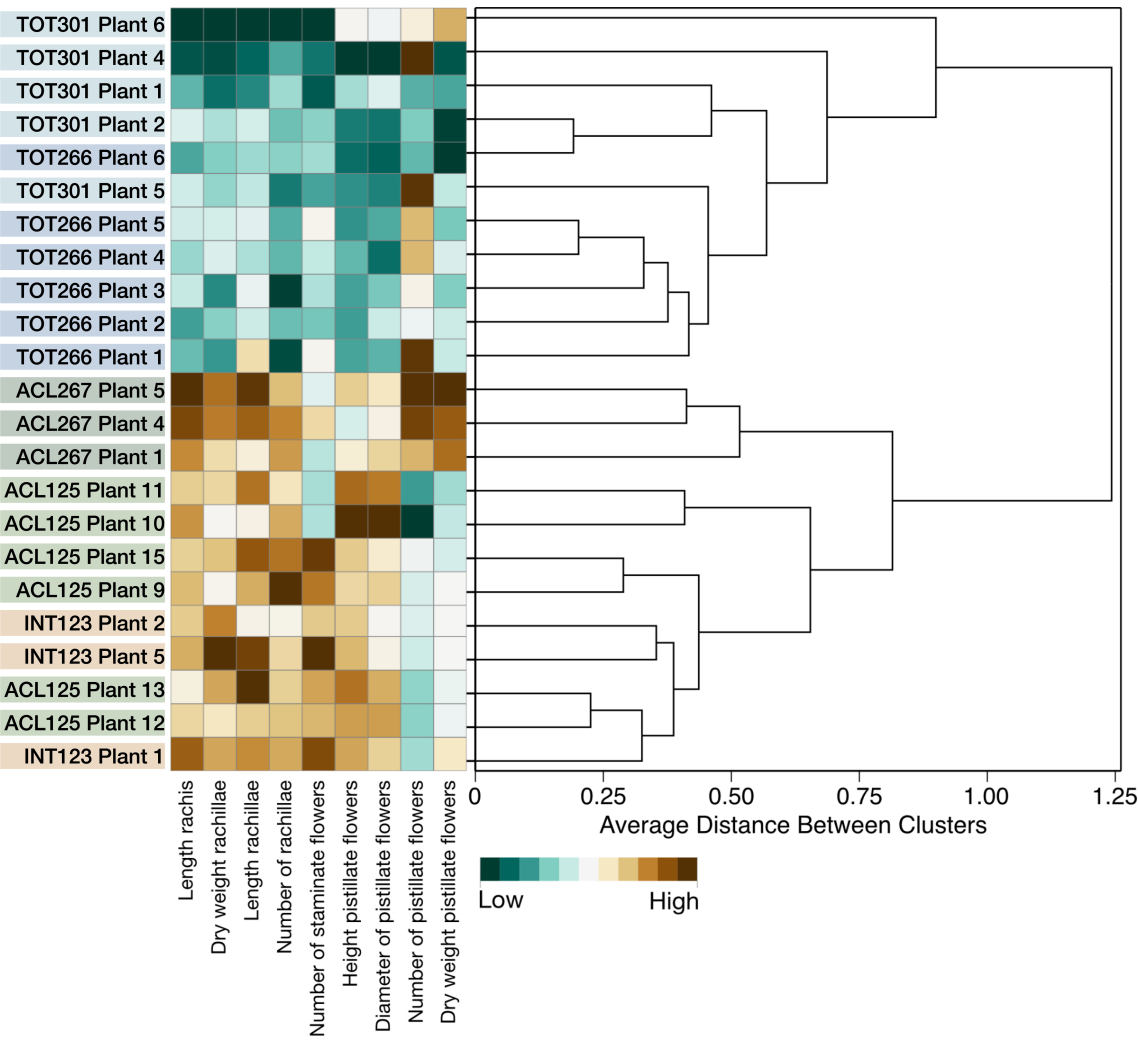
The unweighted pair group method with arithmetic mean (UPGMA) dendrogram estimated according to the Gower distance for nine quantitative inflorescence and flower traits. Low = minimum measured, High = maximum measured.

The evaluation of the accessions revealed species‐specific morphological and biometric variation of the inflorescences and floral structures. Cluster analysis based on all individual flowering palm trees divided the accessions into two groups (Figure 7), one composed of solely the accessions of *A. totai* and the other containing the accessions of *A. aculeata* and *A. intumescens*.

The division into two groups reflects the differentiation of inflorescence architecture and flower traits. The first group, characterized by traits associated with *A. aculeata* and *A. intumescens*, includes long rachises, a high number of heavy and long rachillae, and a low number of large pistillate flowers. On the contrary, the second group, associated with *A. totai*, exhibits short rachises, fewer rachillae, and a higher number of smaller pistillate flowers. The length of the rachis, the number and size of rachillae, and the size of pistillate flowers are positively correlated (Figure 4). In the two‐way clustering analysis, these groups are visually distinguishable by the division of colors, with one group showing high values for these traits and the other showing low values, thereby reinforcing their combined classification.

A differentiation of TOT266 and TOT301 was not possible. Among *A. aculeata* and *A. intumescens*, ACL125 and INT123 could not be distinguished based on their inflorescences and floral structure characteristics.

## 4 DISCUSSION

The delimitation of the species *A. aculeata*, *A. intumescens*, and *A. totai* relies heavily on morphological characteristics of the stem and fruits and the information on geographical distribution. The identification of the species is hindered by overlapping characteristics and a high phenotypic diversity of the intraspecific wild populations. In addition, hybridization of species can occur in areas where they coexist (Díaz et al., 2021), particularly between the widely distributed *A. aculeata* and either *A. totai*, endemic to the South‐West of Brazil, Paraguay, and Argentina, or *A. intumescens*, restricted to the North‐East of Brazil. The framework of viewing the currently accepted species as hypothetical but distinct entities allows for a more exhaustive assessment of the biological diversity (morphoanatomical and genetic) of the wild populations and the potential species boundaries while allowing a quantitative data‐driven debate on the taxonomic status (Galtier, 2019; Hey et al., 2003). The characterization of interspecies and intraspecies morphological differences in the inflorescence architecture and floral structures of *Acrocomia* populations could contribute to the taxonomic delimitation of the species and be an informative tool for domestication initiatives and breeding programs.

Notably, the study of all 382 inflorescences studied showed floral structures consistent with those common to palm species of the subfamily Arecoideae. A denotative floral characteristic of the Arecoideae are the triads, a cluster of a central pistillate flower flanked by two staminate flowers (Tomlinson et al., 2011). The studied rachillae generally followed the sequence of floral structures as described for *Acrocomia* among others by Dransfield et al. (2008), Mazzottini‐dos‐Santos et al. (2015), and Scariot et al. (1991), bearing triads at their basal end and staminate flowers toward the tip. However, differences in the sequence of floral structures at the basal end were a common observation. Floral variations on the flower or inflorescence level are frequent in the palm family and are also an indication of the high environmental susceptibility of the Arecaceae to factors, such as temperature oscillations, water availability, and high vapor pressure deficit (Brancalion et al., 2018; Göldel et al., 2015; Hill et al., 2023; Souza et al., 2023). We observed floral groups reduced from triads by abortion of either pistillate or staminate flowers, i.e., solitary staminate or pistillate flowers and staminate dyads in between the triads.

The most surprising observation, though, was the presence of second‐order rachillae. As far as we know, this study is the first to document the existence of second‐order rachillae in *Acrocomia* species. These second‐order rachillae were only discovered in certain individual palms of *A. totai* accessions, in particular TOT266, which emphasizes not only the phenotypic diversity of the genus but also the phenotypic variation within wild populations.

In their comprehensive study on floral structures in *A. aculeata*, Mazzottini‐dos‐Santos et al. (2015) described the rare occurrence of two deviant floral structures: pairs of pistillate flowers and staminate flowers with well‐developed infertile carpels. Staminate flowers with a well‐developed infertile carpel, where stamens transformed into carpel‐like structures, were also found in the present study in isolated staminate flowers of staminate dyads, triads, and in the apical region of the rachillae. Unlike Mazzottini‐dos‐Santos et al. (2015), we did not observe this vestigial phenotype in *A. aculeata*, but only in the accessions of *A. totai*. The staminate flowers with a well‐developed infertile carpel resemble the mantled flower phenotype in oil palm (Mazzottini‐dos‐Santos et al., 2015), a soma‐clonal variation caused by epigenetic homeotic changes in the floral ABC model (Adam et al., 2005, 2007). The mantled phenotype affects both genders where fertile or sterile androecia are transformed into carpel‐like structures (Adam et al., 2005, 2007) resulting in sterile staminate flowers with carpels and partially or totally sterile pistillate flowers with supernumerary carpels (Adam et al., 2005; Beulé et al., 2011). A phenotype expressing similar structurally abnormal flowers was found in date palm (Cohen et al., 2004). These phenotypes are observed in oil palm and date palm plants regenerated through micropropagation by somatic embryogenesis (Beulé et al., 2011; Cohen et al., 2004). In both crops, these vestigial phenotypes are present in pistillate flowers, which leads to a reduction in yield. In contrast, the staminate flowers with a well‐developed infertile carpel occur spontaneously in wild populations of *Acrocomia*. The causes of this phenotype in wild populations of *Acrocomia* are unknown, and pistillate flowers with supernumerary carpels are yet to be observed in *Acrocomia*. Further studies on the underlying causes of the development of the mantled phenotypes could be of interest, as in vitro propagation of *Acrocomia* is necessary to facilitate breeding programs and the generation of planting material.

In our field observations, the distinct visual differences between inflorescences of *A. totai* and those of *A. aculeata* and *A. intumescens* were unmistakable. Moreover, the application of the Gower distance analysis to inflorescence architecture and floral traits (Figure 7) resulted in the separation of the accessions into two large clusters: the accessions of *A. totai* contrasted with those of *A. aculeata* and *A. intumescens*. The rachis and rachillae of an inflorescence are the branching scaffold providing physical support for the flowers and subsequently the fruits (Harder & Prusinkiewicz, 2013). The three‐dimensional (3D) geometry of an *Acrocomia* inflorescence is characterized by the length of the rachis and the number and length of rachillae. A third important trait, not considered in this study, is the branching angle (Harder & Prusinkiewicz, 2013). The investigated accessions of *A. totai* presented a shorter rachis length and rachillae length, and a lower number of rachillae than those of *A. aculeata* and *A. intumescens*. This is consistent with the findings of Silva et al. (2020) who compared wild populations of *A. totai* and *A. aculeata* based on 41 morphoagronomic descriptors including inflorescence and infructescence architecture. The short rachis and rachillae of *A. totai* lead to a more compact and rigid appearance of the inflorescences.

Furthermore, the inflorescences were rarely pendulous, but more often nested in the leaf sheath of their corresponding leaf without a visible peduncle. By comparison, in both *A. aculeata* and *A. intumescens* the inflorescences drooped between the leaves, along the trunk, due to a visible peduncle. Moreover, the rachillae dangled from the rachis, resulting in loose appearing inflorescences. However, visual discrimination was not possible between the inflorescences of *A. aculeata* and *A. intumescens*. Compared to studies including inflorescence traits of *A. totai* (Silva et al., 2020; Vianna et al., 2021) and *A. aculeata* (Mazzottini‐dos‐Santos et al., 2015; Silva et al., 2020), for both species the length of the rachis of the inflorescences in the present study was shorter and the number of rachillae per inflorescence was lower, whereas the rachillae were longer.

The discrepancy of the biometric traits, particularly in pistillate flower number and size, and number of rachillae between the accessions of *Acrocomia totai* (TOT266 and TOT301: high number of small‐sized pistillate flowers) to the other accessions (ACL125 and INT123: low number of larger pistillate flowers), could be attributed to resource allocation and reproductive strategies (Kettle et al., 2011). Mazzottini‐dos‐Santos et al. (2015) observed a high variability in the biometry of *Acrocomia aculeata* flowering traits but could not distinguish between the environmental effect of the collection areas in northern Minas Gerais and the genetic variability. Since the accessions in the current study are grown at the same site and exposed to similar environmental conditions and management, we hypothesize that the observed discrepancy may be caused by the evolutionary adaption of different reproductive strategies including pollinator attraction strategies and reproductive success, to the environmental conditions of their natural habitat. However, the complexity of plant–environment interactions and their impact on the phenotypic biometry and genetic variability in *Acrocomia* are poorly understood. Some traits contributing to reproductive success, such as fruit size, appear to be more environmentally susceptible than the number of producing infructescences, for instance (Coelho et al., 2017). Furthermore, it remains uncertain whether the traits of any accession cause trade‐offs that impact pollinator attraction and reproductive success, particularly since inflorescence architecture and flower display play crucial roles in pollinator–plant interactions (Harder & Prusinkiewicz, 2013; Prusinkiewicz et al., 2007).

The *Acrocomia totai* and ACL267 accessions have a high number of pistillate flowers, which may decrease the chance of each flower being visited by pollinators (Kettle et al., 2011). However, the shorter rachillae and densely arranged pistillate flowers could mitigate this effect. Additionally, it is unclear if the smaller overall inflorescence display of *Acrocomia totai* accessions, with their less visible bracts, adversely affects pollinator attraction (Harder et al., 2004; Harder & Prusinkiewicz, 2013), potentially resulting in reduced pollination success in plantations where different *Acrocomia* species are cultivated together. Fruit set in *Acrocomia aculeata* and *Acrocomia totai* is generally low and varies widely among infructescences (Scariot et al., 1995; Silva et al., 2020). In *Acrocomia aculeata*, no relationship was found between the number of pistillate flowers, the number of rachillae, and the number of fruits produced, indicating that female reproductive success is majorly dependent on the flowering synchronicity of the inflorescences and the fruit maturation period (Scariot et al., 1995). Since *Acrocomia* fruit maturation occurs supra‐annually and takes 12 to 14 months, the fruit set is influenced by factors such as seed predation and adverse climate conditions, rather than just pollination success (Montoya et al., 2016; Scariot et al., 1995).

Despite variations in biometrics among the six accessions, the inflorescences consistently displayed traits associated with beetle pollination. These traits include yellow to cream‐colored inflorescences with small staminate and pistillate flowers, short‐lived staminate flowers clustered at the apical end of the rachillae, protogyny, and the absence of nectaries (Henderson, 1986). The primary pollinators of *Acrocomia* are derelomine flower weevils (*Andranthobius* spp., Derelomini, Curculionidae) and mystropine sap beetles (*Mystrops* spp., Mystropini, Nitidulidae; Carreño‐Barrera et al., 2021; Scariot et al., 1991), both associated with a wide range of neotropical palms as a result of co‐evolutionary processes (Henderson, 1986). These pollinators are present in high numbers on *Acrocomia* inflorescences upon bract opening and remain until the staminate flowers wither (Carreño‐Barrera et al., 2021; Scariot et al., 1991) The petals of the *Acrocomia* flowers contain specialized osmophores (Mazzottini‐dos‐Santos et al., 2015) that emit volatile olfactory semiochemicals attracting insects upon bract opening (Maia et al., 2018, 2020). The short period during which the pistillate flowers are receptive does not provide food rewards for insects. However, the apical spatial arrangement and the anthesis of the staminate flowers offer potential oviposition sites, shelter, and pollen rewards to pollinators (Carreño‐Barrera et al., 2021; Henderson, 1986; Scariot et al., 1991). The characteristics of the inflorescences and flowers also attract the cyclocephaline scarab beetles (*Cyclocephala* spp., Melolonthidae, Cyclocephalini; Gonçalves et al., 2020; Maia et al., 2020). These beetles are considered pests in Acrocomia, as they feed on flower tissue and cause pistillate flower abortion (Oliveira & Ávila, 2011). However, the extent of yield damage caused by *Cyclocephala* spp. is currently unknown. Future research into insect–plant dynamics, coupled with the recognition of phenotypic distinctions within the genus, is crucial for understanding these ecological interactions and establishing feasible integrated pest management strategies for *Acrocomia* plantations.

The divergence in biometric traits found in this study supports the hypotheses of a taxonomic distinction of *A. totai* as an individual taxon as previously demonstrated by the geographic distribution (de Lima et al., 2018; Lorenzi et al., 2010), fruit biometry (Madeira et al., 2024; Vianna, Berton, et al., 2017), leaf anatomy (Vianna, Carmelo‐Guerreiro, et al., 2017), and genetic structure of the genus (De Lima et al., 2020; Díaz et al., 2021;Lanes et al., 2015; Madeira et al., 2024). In contrast, *A. intumescens* could not be delimited from *A. aculeata* based on the inflorescence architecture and flower traits. Besides its geographic restriction to the North‐East of Brazil, the swelling of the trunk is the only morphological characteristic used to distinguish *A. intumescens* from *A. aculeata* (Díaz et al., 2021; Lorenzi et al., 2010). However, the swelling of the trunk is highly dependent on environmental factors (Tomlinson, 1990). The attempts of previous studies to delimit *A. intumescens* showed an overlap with *A. aculeata* in fruit biometry (Vianna, Berton, et al., 2017), leaf morphoanatomy (Vianna, Carmelo‐Guerreiro, et al., 2017), and genetic structure (Díaz et al., 2021). Furthermore, information on the morphological diversity of wild populations of *A. intumescens* is scarce and most studies conducted on the species focus on fruit biometry and oil composition. The similarities between *Acrocomia aculeata* populations from Minas Gerais and *Acrocomia intumescens* are likely caused by ongoing gene flow by seed spreading facilitated by the São Francisco River, which originates in central Minas Gerais and crosses four northeastern Brazilian states (Lanes et al., 2015; Madeira et al., 2024). Furthermore, the geographic distribution of the two species overlaps in the Brazilian federal states of Ceará and the northern regions of the federal state of Bahia. The Serra da Mantiqueira, a mountain range located in the Brazilian states of São Paulo, Rio de Janeiro, and Minas Gerais, acts potentially as a geographical barrier that separates the populations of *Acrocomia*. This barrier may limit the dispersal of seeds and pollen between the *Acrocomia aculeata* populations in southeastern Brazil and the *Acrocomia totai* populations in Mato Grosso do Sul. The observed separation of *Acrocomia totai* from *Acrocomia aculeata* and *Acrocomia intumescens* accessions in this study and former studies (De Lima et al., 2020; Díaz et al., 2021; Lanes et al., 2015; Madeira et al., 2024; Vianna, Berton, et al., 2017; Vianna, Carmelo‐Guerreiro, et al., 2017) may indicate an ongoing or established speciation process. The seed dispersal of *Acrocomia totai* populations to Paraguay and Argentina was probably facilitated by the Paraguay and Paraná rivers, originating in southwestern Brazil and flowing through Paraguay, Argentina (as Paraná River) to the Rio de la Plata between Argentina and Uruguay. Our results on the lack of a species delimitation of *A. intumescens* should be taken with caution as we had only one accession of *A. intumescens* at our disposal to do this study. We also want to emphasize the importance of further research on the morphological diversity of *A. intumescens*. The species shows a high potential for economic exploitation based on its productivity and oil composition (Barbosa da Silva et al., 2021; Bora & Rocha, 2004). So, it is a valuable resource for domestication initiatives and breeding programs and for agronomic expansion of *Acrocomia* cultivation to the North‐East of Brazil.

Though not endangered taxa, *Acrocomia* occurs mainly in areas with high anthropogenic disturbance, and its natural habitats are highly fragmented, which may decrease the genetic diversity of the natural populations as gene flow between populations is restricted (Clement et al., 2005; Coelho et al., 2018; Navarro‐Cascante et al., 2023). This is particularly the case as the major pollinators are small‐bodied insects with short flight distances (Lanes et al., 2015). This makes conservation efforts crucial for preserving *Acrocomia*'s genetic diversity (Abreu et al., 2012), considering its ecological and agronomic value. Given the environmental impact on the phenotypical plasticity of *Acrocomia* (Ciconini et al., 2013; Coelho et al., 2018; Machado et al., 2015), it is essential to question whether phenotypical variability correlates with genetic diversity. The most feasible approach to conserving critical biodiversity is currently the population‐based conservation efforts, considering the available knowledge on the genus *Acrocomia*. Notably for *Acrocomia totai*, and probably also for *Acrocomia intumescens*, the conservation of their genetic pool is essential as natural populations are threatened by the expansion of commercial plantations of *Acrocomia aculeata* to the South‐West and North‐East of Brazil (Vianna et al., 2021). Exchanging and planting identical genetic material from diverse wild populations in different *Acrocomia* living germplasm banks is crucial for comprehensive comparative studies. Maintaining these accessions at the same site over several years allows for studying phenotypic and annual variation under consistent edaphoclimatic and management conditions (Migicovsky et al., 2019). Simultaneously, exchanging material between germplasm banks enables comparisons of the individual accessions at different sites, providing insights into the impact of varying environmental conditions. This approach would contribute to distinguishing between environmental and genetic effects on traits, such as characteristics of the reproductive biology, to gain a better understanding of reproductive success and facilitate systematics, genetic, and ecological studies on *Acrocomia*'s diversity.

Our assessment of *Acrocomia*'s inflorescence and flower traits showed a major biometric variability between and within accessions, including the existence of second‐order branching. Despite the resulting overlap of the quantitatively assessed characteristics of the accessions, the study showed a distinction between *A. totai* from *A. aculeata* and *A. intumescens*, suggesting an ongoing speciation process. These findings highlight the potential usefulness of inflorescence and flower traits as an additional tool for the taxonomic resolution of these three species. Exploring a wider range of wild populations and accessions from germplasm collections in future studies would provide deeper insights into the intraspecific and interspecific biological diversity of these traits. The high phenotype variability observed provides further evidence of the importance to acknowledge the biological diversity of the wild *Acrocomia* populations in future studies and to correctly identify the taxonomic entity of wild populations and accessions in germplasm collections based on the knowledge present.

## AUTHOR CONTRIBUTIONS

**Catherine Meyer:** Conceptualization (lead); formal analysis (lead); investigation (lead); visualization (lead); writing – original draft (lead); writing – review and editing (equal). **Thomas Hilger:** Conceptualization (supporting); supervision (supporting); writing – review and editing (equal). **Kalcida Naomi Kuki:** Writing – review and editing (equal). **Sérgio Yoshimitsu Motoike:** Resources (lead); supervision (supporting); writing – review and editing (equal). **Georg Cadisch:** Conceptualization (supporting); supervision (lead); writing – review and editing (equal).

## CONFLICT OF INTEREST STATEMENT

The authors declare that they have no conflict of interest.

## Supporting information


Appendix Figure 1

Appendix Tables

## Data Availability

All data files including metadata and the raw data on inflorescence architecture, floral structure sequence, flower biometry, and rachillae traits are accessible in the Harvard Dataverse under the following URL: https://doi.org/10.7910/DVN/FSTGTE.

## APPENDIX A

**TABLE A1:** Number of staminate flowers per rachilla of a total *N* of rachillae split by the position of the rachillae within the inflorescence (Base, Middle, Apex), assessed in both flowering seasons of 2019/2020 and 2021/2022 at the BAG‐Macaúba, Araponga, MG, Brazil.

Accession	Rachilla position within inflorescence	*N* rachillae	Number staminate flowers per rachilla			
			Median	Min	Max	MAD
INT123	Base	27	496	360	624	55
	Middle	27	438	332	506	21
	Apex	24	389	290	506	19
ACL267	Base	44	406	303	529	37
	Middle	41	313	258	431	27
	Apex	41	205	108	286	31
ACL125	Base	90	480	285	666	46
	Middle	90	417	217	513	41
	Apex	89	329	198	440	31
TOT266	Base	145	320	180	454	36
	Middle	133	311	156	441	38
	Apex	140	248	122	368	33
TOT301	Base	116	242	138	341	35
	Middle	103	204	116	339	33
	Apex	104	168	68	296	39

Abbreviation: MAD, median absolute deviation.

**TABLE A2:** Length of rachillae in centimeters (cm) of a total *N* of rachillae split by the position of the rachillae within the inflorescence (Base, Middle, Apex), assessed in both flowering seasons of 2019/2020 and 2021/2022 at the BAG‐Macaúba, Araponga, MG, Brazil.

Accession	Rachilla position within inflorescence	*N* rachillae	Length of rachillae in cm			
			Median	Min	Max	MAD
INT123	Base	27	297	224	357	27
	Middle	27	275	239	327	17
	Apex	27	218	172	274	18
ACL267	Base	44	343	263	478	25
	Middle	44	297	196	357	18
	Apex	44	218	94	305	48
ACL125	Base	102	299	188	376	27
	Middle	102	283	196	347	21
	Apex	104	238	171	294	30
TOT266	Base	154	263	147	355	23
	Middle	153	241	159	353	23
	Apex	155	158	86	235	29
TOT301	Base	131	193	90	344	41
	Middle	131	183	120	300	40
	Apex	132	122	60	201	34

Abbreviation: MAD, median absolute deviation.

## References

[ece370053-bib-0001] Abreu, A. G. , Priolli, R. H. G. , Azevedo‐Filho, J. A. , Nucci, S. M. , Zucchi, M. I. , Coelho, R. M. , & Colombo, C. A. (2012). The genetic structure and mating system of *Acrocomia aculeata* (Arecaceae). Genetics and Molecular Biology, 35(1), 116–121. 10.1590/S1415-47572012005000002 PMC3313499 22481883

[ece370053-bib-0002] Adam, H. , Jouannic, S. , Escoute, J. , Duval, Y. , Verdeil, J.‐L. , & Tregear, J. W. (2005). Reproductive developmental complexity in the African oil palm (*Elaeis guineensis*, Arecaceae). American Journal of Botany, 92(11), 1836–1852. 10.3732/ajb.92.11.1836 21646101

[ece370053-bib-0003] Adam, H. , Jouannic, S. , Orieux, Y. , Morcillo, F. , Richaud, F. , Duval, Y. , & Tregear, J. W. (2007). Functional characterization of MADS box genes involved in the determination of oil palm flower structure. Journal of Experimental Botany, 58(6), 1245–1259. 10.1093/jxb/erl263 17339652

[ece370053-bib-0004] Alvares, C. A. , Stape, J. L. , Sentelhas, P. C. , de Moraes Gonçalves, J. L. , & Sparovek, G. (2013). Köppen's climate classification map for Brazil. Meteorologische Zeitschrift, 22(6), 711–728. 10.1127/0941-2948/2013/0507

[ece370053-bib-0005] Baker, W. J. , & Dransfield, J. (2016). Beyond *Genera Palmarum*: Progress and prospects in palm systematics. Botanical Journal of the Linnean Society, 182(2), 207–233. 10.1111/boj.12401

[ece370053-bib-0006] Barbosa da Silva, R. B. d. , Vieira da Silva‐Júnior, E. , Trigueiros, L. M. B. d. M. , Santos, R. H. G. d. , Aquino, J. d. S. , Campos, A. R. N. , & de Oliveira, A. F. M. (2021). “Macaíba”, an emerging oil crop: Nutritional evaluation of the pulp and kernel fruits from semi‐arid and coastal zone of northeast Brazil. Journal of Agronomy and Crop Science, 207(1), 139–147. 10.1111/jac.12435

[ece370053-bib-0007] Beulé, T. , Camps, C. , Debiesse, S. , Tranchant, C. , Dussert, S. , Sabau, X. , Jaligot, E. , Alwee, S. S. R. S. , & Tregear, J. W. (2011). Transcriptome analysis reveals differentially expressed genes associated with the mantled homeotic flowering abnormality in oil palm (*Elaeis guineensis*). Tree Genetics and Genomes, 7(1), 169–182. 10.1007/s11295-010-0323-9

[ece370053-bib-0008] Bora, P. S. , & Rocha, R. V. M. (2004). Macaíba palm: Fatty and amino acids composition of fruits. Ciencia y Tecnologia Alimentaria, 4(3), 158–162. 10.1080/11358120409487755

[ece370053-bib-0009] Brancalion, P. H. S. , Oliveira, G. C. X. , Zucchi, M. I. , Novello, M. , van Melis, J. , Zocchi, S. S. , Chazdon, R. L. , & Rodrigues, R. R. (2018). Phenotypic plasticity and local adaptation favor range expansion of a neotropical palm. Ecology and Evolution, 8(15), 7462–7475. 10.1002/ece3.4248 30151163 PMC6106193

[ece370053-bib-0010] Cardoso, A. , Laviola, B. G. , Santos, G. S. , de Sousa, H. U. , de Oliveira, H. B. , Veras, L. C. , Ciannella, R. , & Favaro, S. P. (2017). Opportunities and challenges for sustainable production of *A. aculeata* through agroforestry systems. Industrial Crops and Products, 107, 573–580. 10.1016/j.indcrop.2017.04.023

[ece370053-bib-0011] Carreño‐Barrera, J. , Maia, A. C. D. , Colombo, C. A. , & Núñez‐Avellaneda, L. A. (2021). Co‐pollination, constancy, and efficiency over time: Small beetles and the reproductive success of *Acrocomia aculeata* (Arecaceae) in the Colombian Orinoquia. Botany Letters, 168(3), 395–407. 10.1080/23818107.2021.1893215

[ece370053-bib-0012] Ciconini, G. , Favaro, S. P. , Roscoe, R. , Miranda, C. H. B. , Tapeti, C. F. , Miyahira, M. A. M. , Bearari, L. , Galvani, F. , Borsato, A. V. , Colnago, L. A. , & Naka, M. H. (2013). Biometry and oil contents of *Acrocomia aculeata* fruits from the Cerrados and Pantanal biomes in Mato Grosso do Sul, Brazil. Industrial Crops and Products, 45, 208–214. 10.1016/j.indcrop.2012.12.008

[ece370053-bib-0013] Clement, C. R. , Lleras, E. , & van Leeuwen, J. (2005). O potencial das palmeiras tropicais no Brasil acertos e fracassos das últimas décadas. Agrociencia, 9, 67–71.

[ece370053-bib-0014] Coelho, N. H. P. , Tambarussi, E. V. , Aguiar, B. I. , Roque, R. H. , Portela, R. M. , Braga, R. C. , Sanson, D. , Silva, R. A. R. , Ferraz, E. M. , Moreno, M. A. , Kageyama, P. Y. , & Gandara, F. B. (2018). Understanding genetic diversity, spatial genetic structure, and mating system through microsatellite markers for the conservation and sustainable use of *Acrocomia aculeata* (Jacq.) Lodd. Ex Mart. Conservation Genetics, 19(4), 879–891. 10.1007/s10592-018-1061-z

[ece370053-bib-0015] Coelho, R. M. , Da Costa, C. F. , de Azevedo Filho, J. A. , Chorfi Berton, L. H. , & Colombo, C. A. (2017). Non‐biotic factors determining plasticity of the prospective oil‐rich macaúba palm (*Acrocomia aculeata*). Agroforestry Systems, 93, 771–782.

[ece370053-bib-0016] Cohen, Y. , Korchinsky, R. , & Tripler, E. (2004). Flower abnormalities cause abnormal fruit setting in tissue culture‐propagated date palm (*Phoenix dactylifera* L.). The Journal of Horticultural Science and Biotechnology, 79(6), 1007–1013. 10.1080/14620316.2004.11511853

[ece370053-bib-0017] Colombo, C. A. , Chorfi Berton, L. H. , Diaz, B. G. , & Ferrari, R. A. (2018). Macaúba: A promising tropical palm for the production of vegetable oil. Oilseeds and Fats Crops and Lipids, 25(1), D108. 10.1051/ocl/2017038

[ece370053-bib-0018] de Lima, N. E. , Carvalho, A. A. , Meerow, A. W. , & Manfrin, M. H. (2018). A review of the palm genus *Acrocomia*: Neotropical green gold. Organisms Diversity and Evolution, 18(2), 151–161. 10.1007/s13127-018-0362-x

[ece370053-bib-0019] De Lima, N. E. , Meerow, A. W. , & Manfrin, M. H. (2020). Genetic structure of two *Acrocomia* ecotypes (Arecaceae) across Brazilian savannas and seasonally dry forests. Tree Genetics and Genomes, 16(4), 56. 10.1007/s11295-020-01446-y

[ece370053-bib-0020] Díaz, B. G. , Zucchi, M. I. , Alves‐Pereira, A. , de Almeida, C. P. , Moraes, A. C. L. , Vianna, S. A. , Azevedo‐Filho, J. , & Colombo, C. A. (2021). Genome‐wide SNP analysis to assess the genetic population structure and diversity of *Acrocomia* species. PLoS One, 16(7), e0241025. 10.1371/journal.pone.0241025 34283830 PMC8291712

[ece370053-bib-0021] Dransfield, J. , Uhl, N. W. , Asmussen, C. B. , Baker, W. J. , Harley, M. M. , & Lewis, C. E. (2008). Genera palmarum: The evolution and classification of palms (1st ed.). Kew Publishing Royal Botanical Garden.

[ece370053-bib-0022] *Flora e Funga do Brasil* . (2024, May 30). Flora e Funga Do Brasil. https://floradobrasil.jbrj.gov.br/consulta/.

[ece370053-bib-0023] Freitas de Lima, F. , Lescano, C. H. , & Pires de Oliveira, I. (Eds.). (2021). Fruits of the Brazilian Cerrado: Composition and functional benefits. Springer International Publishing. 10.1007/978-3-030-62949-6

[ece370053-bib-0024] Galtier, N. (2019). Delineating species in the speciation continuum: A proposal. Evolutionary Applications, 12(4), 657–663. 10.1111/eva.12748 30976300 PMC6439491

[ece370053-bib-0025] Göldel, B. , Kissling, W. D. , & Svenning, J.‐C. (2015). Geographical variation and environmental correlates of functional trait distributions in palms (Arecaceae) across the New World: Functional traits in New World palms. Botanical Journal of the Linnean Society, 179(4), 602–617. 10.1111/boj.12349

[ece370053-bib-0026] Gonçalves, J. A. , Grossi, P. C. , Togni, P. H. B. , Oliveira, C. M. , & Frizzas, M. R. (2020). The genus Cyclocephala Dejean (coleoptera: Scarabaeidae: Dynastinae) in Brazil: Diversity and spatio‐temporal distribution. Journal of Insect Conservation, 24(3), 547–559. 10.1007/s10841-020-00230-6

[ece370053-bib-0027] Harder, L. D. , Jordan, C. Y. , Gross, W. E. , & Routley, M. B. (2004). Beyond floricentrism: The pollination function of inflorescences. Plant Species Biology, 19(3), 137–148. 10.1111/j.1442-1984.2004.00110.x

[ece370053-bib-0028] Harder, L. D. , & Prusinkiewicz, P. (2013). The interplay between inflorescence development and function as the crucible of architectural diversity. Annals of Botany, 112(8), 1477–1493. 10.1093/aob/mcs252 23243190 PMC3828939

[ece370053-bib-0029] Henderson, A. (1986). A review of pollination studies in the Palmae. The Botanical Review, 52(3), 221–259. 10.1007/BF02860996

[ece370053-bib-0030] Henderson, A. (1995). The palms of the Amazon. Oxford University Press.

[ece370053-bib-0031] Hey, J. , Waples, R. S. , Arnold, M. L. , Butlin, R. K. , & Harrison, R. G. (2003). Understanding and confronting species uncertainty in biology and conservation. Trends in Ecology and Evolution, 18(11), 597–603. 10.1016/j.tree.2003.08.014

[ece370053-bib-0032] Hill, A. , Jiménez, M. F. T. , Chazot, N. , Cássia‐Silva, C. , Faurby, S. , Herrera‐Alsina, L. , & Bacon, C. D. (2023). Apparent effect of range size and fruit colour on palm diversification may be spurious. Journal of Biogeography, 50(10), 1724–1736. 10.1111/jbi.14683

[ece370053-bib-0033] Kettle, C. J. , Maycock, C. R. , Ghazoul, J. , Hollingsworth, P. M. , Khoo, E. , Sukri, R. S. H. , & Burslem, D. F. R. P. (2011). Ecological implications of a flower size/number trade‐off in tropical Forest trees. PLoS One, 6(2), e16111. 10.1371/journal.pone.0016111 21408110 PMC3052255

[ece370053-bib-0034] Lanes, É. C. M. , Motoike, S. Y. , Kuki, K. N. , de Resende, M. D. V. , & Caixeta, E. T. (2016). Mating system and genetic composition of the macaw palm (*Acrocomia aculeata*): Implications for breeding and genetic conservation programs. Journal of Heredity, 107(6), 527–536. 10.1093/jhered/esw038 27288529

[ece370053-bib-0035] Lanes, E. C. M. , Motoike, S. Y. , Kuki, K. N. , Nick, C. , & Freitas, R. D. (2015). Molecular characterization and population structure of the macaw palm, *Acrocomia aculeata* (Arecaceae), ex situ germplasm collection using microsatellites markers. Journal of Heredity, 106(1), 102–112. 10.1093/jhered/esu073 25425677

[ece370053-bib-0036] Lorenzi, H. , Noblick, L. R. , Kahn, F. , & Ferreira, E. (2010). Flora brasileira. Lorenzi: Arecaceae (palmeiras). Instituto Plantarum.

[ece370053-bib-0037] Machado, W. , Guimarães, M. F. , Lira, F. F. , Santos, J. V. F. , Takahashi, L. S. A. , Leal, A. C. , & Coelho, G. T. C. P. (2015). Evaluation of two fruit ecotypes (*totai* and *sclerocarpa*) of macaúba (*Acrocomia aculeata*). Industrial Crops and Products, 63, 287–293. 10.1016/j.indcrop.2014.11.002

[ece370053-bib-0038] Madeira, D. D. C. , Motoike, S. Y. , Simiqueli, G. F. , Kuki, K. N. , De Melo Goulart, S. , Rigolon, T. C. B. , Nogueira, P. T. S. , Da Silva Castro, A. , & De Oliveira Couto, E. G. (2024). Phenotypic characterization and genetic diversity of macaúba (*Acrocomia aculeata*) accessions based on oil attributes and fruit biometrics. Genetic Resources and Crop Evolution. 10.1007/s10722-024-01856-0

[ece370053-bib-0039] Maia, A. C. D. , Reis, L. K. , Navarro, D. M. D. A. F. , Aristone, F. , Colombo, C. A. , Carreño‐Barrera, J. , Núñez‐Avellaneda, L. A. , & Santos, G. K. N. (2020). Chemical ecology of *Cyclocephala forsteri* (Melolonthidae), a threat to macaúba oil palm cultivars (*Acrocomia aculeata*, Arecaceae). Journal of Applied Entomology, 144(1–2), 33–40. 10.1111/jen.12699

[ece370053-bib-0040] Maia, A. C. D. , Santos, G. K. N. , Gonçalves, E. G. , Navarro, D. M. D. A. F. , & Nuñez‐Avellaneda, L. A. (2018). 2‐Alkyl‐3‐methoxypyrazines are potent attractants of florivorous scarabs (Melolonthidae, Cyclocephalini) associated with economically exploitable neotropical palms (Arecaceae). Pest Management Science, 74(9), 2053–2058. 10.1002/ps.4895 29479808

[ece370053-bib-0041] Mazzottini‐dos‐Santos, H. C. , Ribeiro, L. M. , Mercadante‐Simões, M. O. , & Sant'Anna‐Santos, B. F. (2015). Floral structure in *Acrocomia aculeata* (Arecaceae): Evolutionary and ecological aspects. Plant Systematics and Evolution, 301(5), 1425–1440. 10.1007/s00606-014-1167-9

[ece370053-bib-0042] Menezes Oliveira, D. , de Paula da Costa, J. , Clemente, E. , & Correia da Costa, J. M. (2013). Characterization of grugru palm pulp for food applications. Journal of Food Science and Engineering, 3(2), 107–112.

[ece370053-bib-0043] Migicovsky, Z. , Warschefsky, E. , Klein, L. L. , & Miller, A. J. (2019). Using living germplasm collections to characterize, improve, and conserve Woody perennials. Crop Science, 59(6), 2365–2380. 10.2135/cropsci2019.05.0353

[ece370053-bib-0044] Montoya, S. G. , Motoike, S. Y. , Kuki, K. N. , & Couto, A. D. (2016). Fruit development, growth, and stored reserves in macaúba palm (*Acrocomia aculeata*), an alternative bioenergy crop. Planta, 244(4), 927–938. 10.1007/s00425-016-2558-7 27318823

[ece370053-bib-0045] Morrison, W. R. , Lohr, J. L. , Duchen, P. , Wilches, R. , Trujillo, D. , Mair, M. , & Renner, S. S. (2009). The impact of taxonomic change on conservation: Does it kill, can it save, or is it just irrelevant? Biological Conservation, 142(12), 3201–3206. 10.1016/j.biocon.2009.07.019

[ece370053-bib-0046] Motoike, S. Y. , Carvalho, M. , Pimentel, L. D. , Kuki, K. N. , Paes, J. M. V. , Dias, H. C. T. , & Sato, A. Y. (2013). A cultura da Macaùba: Implantação e manejo de cultivos racionais. Editora UFV.

[ece370053-bib-0047] Navarro‐Cascante, V. , Arnáez‐Serrano, E. , Rojas‐Gómez, M. , González, I. M. , Vargas‐Hernández, G. , Zamora, N. A. , Briceño‐Elizondo, E. , Morales‐Marroquín, J. , Sevilla‐Cortés, P. , Oviedo‐Ulate, J. A. , & Araya‐Valverde, E. (2023). Genetic diversity and structure of *Acrocomia aculeata* (Jacq.) Lodd. Ex Mart. (Arecaceae) using microsatellite DNA markers in Costa Rica. Genetic Resources and Crop Evolution, 70(4), 1277–1288. 10.1007/s10722-022-01501-8

[ece370053-bib-0048] Oliveira, D. A. , Melo Júnior, A. F. , Brandão, M. M. , Rodrigues, L. A. , Menezes, E. V. , & Ferreira, P. R. B. (2012). Genetic diversity in populations of *Acrocomia aculeata* (Arecaceae) in the northern region of Minas Gerais, Brazil. Genetics and Molecular Research, 11(1), 531–538. 10.4238/2012.March.8.1 22535388

[ece370053-bib-0049] Oliveira, H. N. D. , & Ávila, C. J. (2011). Ocorrência de *Cyclocephala forsteri* em *Acrocomia aculeata* . Pesquisa Agropecuária Tropical, 41(2), 293–295. 10.5216/pat.v41i2.8769

[ece370053-bib-0050] Pires, T. P. , dos Santos Souza, E. , Kuki, K. N. , & Motoike, S. Y. (2013). Ecophysiological traits of the macaw palm: A contribution towards the domestication of a novel oil crop. Industrial Crops and Products, 44, 200–210. 10.1016/j.indcrop.2012.09.029

[ece370053-bib-0051] POWO . (2024, May 30). Plants of the World Online. Facilitated by the Royal Botanic Gardens, Kew . http://www.plantsoftheworldonline.org/

[ece370053-bib-0052] Prusinkiewicz, P. , Erasmus, Y. , Lane, B. , Harder, L. D. , & Coen, E. (2007). Evolution and development of inflorescence architectures. Science, 316(5830), 1452–1456. 10.1126/science.1140429 17525303

[ece370053-bib-0053] Rosado, R. D. S. , Rosado, T. B. , Cruz, C. D. , Ferraz, A. G. , Haa Carson Schwartzhaupt Da Conceição, L. D. , & Galveas Laviola, B. (2019). Genetic parameters and simultaneous selection for adaptability and stability of macaw palm. Scientia Horticulturae, 248, 291–296. 10.1016/j.scienta.2018.12.041

[ece370053-bib-0054] Scariot, A. O. , Lleras, E. , & Hay, J. D. (1991). Reproductive biology of the palm *Acrocomia aculeata* in Central Brazil. Biotropica, 23(1), 12. 10.2307/2388683

[ece370053-bib-0055] Scariot, A. O. , Lleras, E. , & Hay, J. D. (1995). Flowering and fruiting Phenologies of the palm Acrocomia aculeata: Patterns and consequences. Biotropica, 27(2), 168. 10.2307/2388992

[ece370053-bib-0056] Silva, P. H. , Vianna, S. A. , Carvalho, C. R. L. , Filho, J. A. A. , & Colombo, C. A. (2020). Divergência genética entra espécies de palmeiras *Acrocomia* Mart. Baseada em descritores morfoagronômicos. Energia na Agricultura, 35(4), 562–577. 10.17224/EnergAgric.2020v35n4

[ece370053-bib-0057] Souza, P. N. S. , Andrade, F. H. P. , Azevedo, A. M. , Nietsche, S. , Ribeiro, L. M. , & Lopes, P. S. N. (2023). Morphoagronomic diversity in *Butia capitata* progenies (Arecaceae). Euphytica, 219(7), 81. 10.1007/s10681-023-03203-3

[ece370053-bib-0058] Tomlinson, P. B. (1990). The structural biology of palms. Oxford Science Publications, Clarendon Press.

[ece370053-bib-0059] Tomlinson, P. B. , Horn, J. W. , & Fisher, J. B. (2011). The anatomy of palms (1st ed.). Oxford University Press.

[ece370053-bib-0060] Vargas‐Carpintero, R. , Hilger, T. , Mössinger, J. , Souza, R. F. , Barroso Armas, J. C. , Tiede, K. , & Lewandowski, I. (2021). *Acrocomia* spp.: Neglected crop, ballyhooed multipurpose palm or fit for the bioeconomy? A review. Agronomy for Sustainable Development, 41(6), 75. 10.1007/s13593-021-00729-5

[ece370053-bib-0061] Vianna, S. A. (2017). A new species of *Acrocomia* (Arecaceae) from Central Brazil. Phytotaxa, 314(1), 45. 10.11646/phytotaxa.314.1.2

[ece370053-bib-0062] Vianna, S. A. , Berton, L. H. C. , Pott, A. , Carmello Guerreiro, S. M. , & Colombo, C. A. (2017). Biometric characterization of fruits and morphoanatomy of the mesocarp of *Acrocomia* species (Arecaceae). International Journal of Biology, 9(3), 78. 10.5539/ijb.v9n3

[ece370053-bib-0063] Vianna, S. A. , Carmelo‐Guerreiro, S. M. , Noblick, L. R. , & Colombo, C. A. (2017). Leaf anatomy of *Acrocomia* (Arecaceae): An additional contribution to the taxonomic resolution of a genus with great economic potential. Plant Systematics and Evolution, 303(2), 233–248. 10.1007/s00606-016-1369-4

[ece370053-bib-0064] Vianna, S. A. , Domenech, H. L. M. , da Silva, R. H. , Colombo, C. A. , & Pott, A. (2021). Morphological characterization and productivity estimates of *Acrocomia totai* Mart. (Arecaceae) – A sustainable alternative of extractivism and cultivation. Revista Brasileira de Fruticultura, 43(1), e730. 10.1590/0100-29452021730

[ece370053-bib-0065] WFO . (2024, May 30). World Flora Online . https://about.worldfloraonline.org

