# Supplementary figures and images for: Biometric variability of inflorescence and flower traits among ex situ accessions of the neotropical oilseed palm Acrocomia Mart
Source: Ecol Evol. 2024 Jul 30;14(7):e70053. doi: 10.1002/ece3.70053 (PMC11287079; doi:10.1002/ece3.70053)

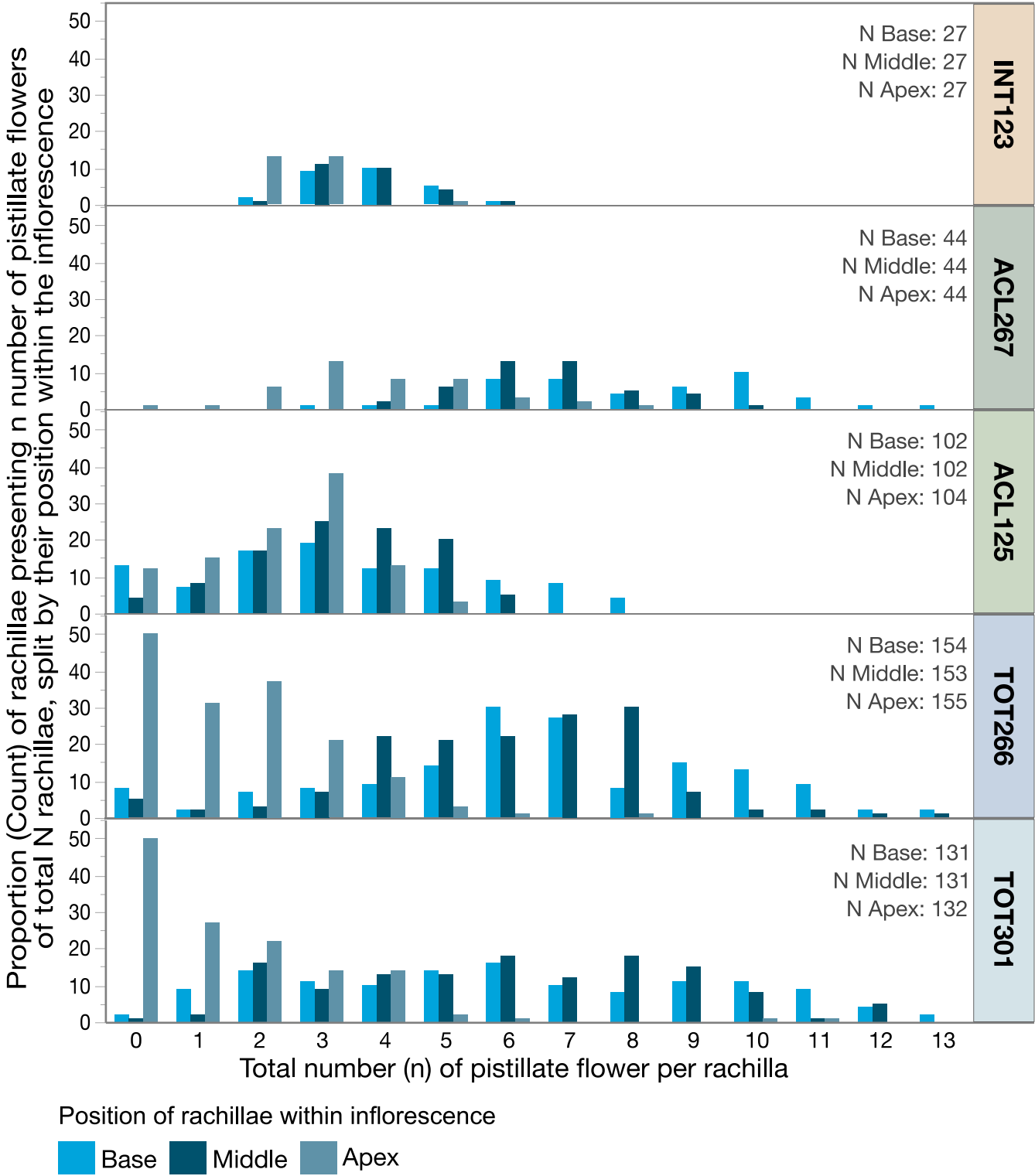

Supplement: Supplementary file 1 — Appendix Figure 1 [file ECE3-14-e70053-s001.pdf]
