# Supplementary material for: Biometric variability of inflorescence and flower traits among ex situ accessions of the neotropical oilseed palm Acrocomia Mart
Source: Ecol Evol. 2024 Jul 30;14(7):e70053. doi: 10.1002/ece3.70053 (PMC11287079; doi:10.1002/ece3.70053)
Supplement: Supplementary file 2 — Appendix Tables [file ECE3-14-e70053-s002.docx]

**Appendix Tables and Figure Legend**

Appendix Table 1. Number of staminate flowers per rachilla of a total N rachillae split by the position of the rachillae within the inflorescence (Base, Middle, Apex), assessed in both flowering seasons of 2019/2020 and 2021/2022 at the BAG-Macaúba, Araponga, MG, Brazil. MAD = Median absolute deviation.

| **Accession** | **Rachilla position within inflorescence** | **N rachillae** | **Number staminate flowers per rachilla** | | | |
| --- | --- | --- | --- | --- | --- | --- |
|  |  |  | **Median** | **Min** | **Max** | **MAD** |
| INT123 | Base | 27 | 496 | 360 | 624 | 55 |
|  | Middle | 27 | 438 | 332 | 506 | 21 |
|  | Apex | 24 | 389 | 290 | 506 | 19 |
| ACL267 | Base | 44 | 406 | 303 | 529 | 37 |
|  | Middle | 41 | 313 | 258 | 431 | 27 |
|  | Apex | 41 | 205 | 108 | 286 | 31 |
| ACL125 | Base | 90 | 480 | 285 | 666 | 46 |
|  | Middle | 90 | 417 | 217 | 513 | 41 |
|  | Apex | 89 | 329 | 198 | 440 | 31 |
| TOT266 | Base | 145 | 320 | 180 | 454 | 36 |
|  | Middle | 133 | 311 | 156 | 441 | 38 |
|  | Apex | 140 | 248 | 122 | 368 | 33 |
| TOT301 | Base | 116 | 242 | 138 | 341 | 35 |
|  | Middle | 103 | 204 | 116 | 339 | 33 |
|  | Apex | 104 | 168 | 68 | 296 | 39 |

Appendix Table 2. Length of rachillae in cm of a total N rachillae split by the position of the rachillae within the inflorescence (Base, Middle, Apex), assessed in both flowering seasons of 2019/2020 and 2021/2022 at the BAG-Macaúba, Araponga, MG, Brazil. MAD = Median absolute deviation

| **Accession** | **Rachilla position within inflorescence** | **N rachillae** | **Length of rachillae in cm** | | | |
| --- | --- | --- | --- | --- | --- | --- |
|  |  |  | **Median** | **Min** | **Max** | **MAD** |
| INT123 | Base | 27 | 297 | 224 | 357 | 27 |
|  | Middle | 27 | 275 | 239 | 327 | 17 |
|  | Apex | 27 | 218 | 172 | 274 | 18 |
| ACL267 | Base | 44 | 343 | 263 | 478 | 25 |
|  | Middle | 44 | 297 | 196 | 357 | 18 |
|  | Apex | 44 | 218 | 94 | 305 | 48 |
| ACL125 | Base | 102 | 299 | 188 | 376 | 27 |
|  | Middle | 102 | 283 | 196 | 347 | 21 |
|  | Apex | 104 | 238 | 171 | 294 | 30 |
| TOT266 | Base | 154 | 263 | 147 | 355 | 23 |
|  | Middle | 153 | 241 | 159 | 353 | 23 |
|  | Apex | 155 | 158 | 86 | 235 | 29 |
| TOT301 | Base | 131 | 193 | 90 | 344 | 41 |
|  | Middle | 131 | 183 | 120 | 300 | 40 |
|  | Apex | 132 | 122 | 60 | 201 | 34 |

Appendix Figure 1. Absolute frequency of the number of pistillate flowers per rachilla, of a total N rachillae split by the position of the rachillae within the inflorescence (Base, Middle, Apex), assessed in both flowering seasons of 2019/2020 and 2021/2022 at the BAG-Macaúba, Araponga, MG, Brazil.
